# Comparative chloroplast genome analyses of 23 species in *Swertia* L. (Gentianaceae) with implications for its phylogeny

**DOI:** 10.3389/fgene.2022.895146

**Published:** 2022-08-31

**Authors:** Lucun Yang, Jingjing Li, Guoying Zhou

**Affiliations:** ^1^ Northwest Institute of Plateau Biology, Chinese Academy of Sciences, Xining, China; ^2^ Key Laboratory of Tibetan Medicine Research, Chinese Academy of Sciences, Xining, China; ^3^ College of Life Science, Qinghai Normal University, Xining, China

**Keywords:** *Swertia*, chloroplast genome, comparative analysis, phylogenetic analysis, repeat sequences

## Abstract

*Swertia* L. is a large genus in the family Gentianaceae. Different chloroplast gene segments have been used to study systematic evolutionary relationships between species of *Swertia* L. However, as gene fragment–based phylogenies lack sufficient resolution, the systematic evolutionary relationships between *Swertia* L. species have remained unclear. We sequenced and annotated the complete chloroplast genomes of four *Swertia* species, namely, *S. bifolia*, *S. tetraptera*, *S. franchetian*, and *S. przewalskii*, using next generation sequencing and the plastid genome annotator tool. The chloroplast genome sequences of 19 additional species of *Swertia* L. were downloaded from the NCBI database and also assessed. We found that all 23 *Swertia* L. species had a similar genetic structure, that is, a ring tetrad structure, but with some clear differences. The chloroplast genomes of the 23 *Swertia* L. species were 149036–153691 bp long, averaging 152385 bp; the genomes contained 134 functional genes: 38 tRNA, eight rRNA, and 88 protein-encoding genes. A comparative analysis showed that chloroplasts genome of *Swertia* was conserved in terms of genome structure, codon preference, and repeat sequences, but it differed in terms of genome sizes, gene contents, and SC/IR boundary. Using *Swertia wolfangiana* as a reference, we found clear divergences in most of the non-coding and intergenic regions of the complete chloroplast genomes of these species; we also found that *rpoC1*, *ccsA*, *ndhI*, *ndhA*, and *rps15* protein-coding genes had large variations. These highly variable hotspots will be useful for future phylogenetic and population genetic studies. Phylogenetic analysis with high bootstrap support showed that *Swertia* L. was not monophyletic. The classification of subgen. *Swertia* and subgen. *Ophelia* was supported by molecular data, which also partly supported the division of sect. *Ophelia*, sect. *Platynema*, sect. *Poephila,* sect*. Swertia*, and sect. *Macranthos*. However, the systematic positions of other groups and species require further exploration. The *Swertia* L formed at 29.60 Ma. Speciation of 10 species occurred in succession after 12 Ma and 13 species occurred in succession after 2.5 Ma. Our analysis provides insight into the unresolved evolutionary relationships of *Swertia* L. species.

## Introduction

*Swertia* L. is a large genus in the family Gentianaceae and is widely distributed in Asia, Africa, and North America, with only a few species found in Europe. There are 170 species of *Swertia* L. plants, divided into 3 subgenera and 11 groups, which include 79 species found in China. These 79 species are most abundant in the Qinghai–Tibetan Plateau (Struwe and Albert, 2002; Von Hagen and Kadereit, 2002; Ho and Liu, 2015). A variety of plants in the *Swertia* L. genus have a long history of medicinal use in China. These plants and their components (such as oleanolic acid) have liver protective, enzyme lowering, anti-inflammatory, cardiotonic, diuretic, and anticancer effects and currently comprise part of an effective drug strategy for the treatment of hepatitis (Liang and Gao, 1979; Chen et al., 1999; Ma et al., 2008). Recent pharmacological studies have shown that plants from this genus can strengthen the heart, lower blood glucose concentration, promote blood circulation, and inhibit testosterone reductase (Li et al., 2007). Thus, a significant amount of attention has been paid to *Swertia* L. because of its extensive pharmacological effects. However, the origins of this genus have been disputed, even at the subgenus and species levels (Chassot et al., 2001; Struwe and Albert, 2002; Von Hagen and Kadereit, 2002; Shi, 2004; Favre et al., 2010; Xi et al., 2014; Cao et al., 2021). Different types of molecular data have been used to study the systematic evolutionary relationships between the species of *Swertia* L. (Chassot et al., 2001; Von Hagen and Kadereit, 2002; Favre et al., 2010; Xi et al., 2014; Cao et al., 2021), all of which have shown that the genus is not monophyletic (Chassot et al., 2001; Von Hagen and Kadereit, 2002; Favre et al., 2010; Xi et al., 2014; Cao et al., 2021). Nevertheless, the systematic relationships within the *Swertia* L. genus have not been well resolved, and there remains great conflict between many molecular systematics studies and the traditional classification system based on morphological traits (Chassot et al., 2001; Von Hagen and Kadereit, 2002; Favre et al., 2010; Xi et al., 2014; Cao et al., 2021). This conflict has mainly been caused by the low resolution of the chloroplast and its gene fragments. Although there are stark differences in sequence variation between genes, the phylogenetic information provided by one or a few gene segments is limited and phylogenetic trees may reflect only the gene trees of the few segments analyzed. Because gene trees are not always equivalent to species trees, they may not represent the true phylogenetic relationships between species. Molecular fragments are an important source of the traits displayed by specific taxa. Although they can provide significant information for the systematic reconstruction of taxa, they cannot truly reflect the real historical evolution of the taxa. Therefore, new techniques have been needed to evaluate the genetic relationships between *Swertia* L. species. At present, molecular markers such as nrDNA, chloroplast DNA, mitochondrial DNA, ISSR, and RAPD were widely used in phylogenetic studies (Hakki et al., 2010; Pikunova et al., 2012; Adams and Schwarzbach, 2013; Liu et al., 2020; Kousteni et al., 2021). Also, RAPD and ISSR were used to access the genetic diversity in *Swertia* L. (Neupane et al., 2017; Chhipi Shrestha et al., 2013). However, as a dominant marker, RAPD and ISSR cannot effectively distinguish heterozygous and homozygous genotypes, so the results are not very reliable when used to study the relationship between species or related genera. In recent years, a comparative analysis of the complete chloroplast genomes of different related species has become a promising method for the study of phylogeny, population dynamics, and species evolution.

Chloroplasts are the descendants of ancient bacteria (early plants and cyanobacteria) and are important organelles for photosynthesis in plants. Thus, they confer on plants the role of producers in the ecological environments of the Earth. Chloroplasts, which are responsible for many metabolic tasks in addition to photosynthesis, are therefore extremely important and energetic organelles in plant cells (Brunkard et al., 2015). Compared with nuclear genomes, chloroplast genomes have the following advantages for a phylogenetic analysis. First, chloroplast genomes have high copy numbers and relatively small complete sequencing sizes, making them suitable for analyzing the evolutionary relationships of plants (McNeal et al., 2006). Second, chloroplasts have a quadripartite structure with 100–130 genes, all of which have highly conserved sequences and competition, making these genomes more conducive to comparison and analysis of evolution and kinship between species (Wicke et al., 2011). Due to its low replacement rate, lack of nucleotide recombination, and uniparental DNA sequence, the chloroplast genome is a key data source for inferring plant phylogeny (Shaw et al., 2005; Chen and Liu, 2008). In recent years, complete chloroplast genomes have been widely used in phylogenetic and genetic relationship analyses of plants, allowing researchers to directly assess the evolutionary relationships between plants (Yang et al., 2016). For example, Yang et al. (2019) reconstructed phylogenetic trees based on whole-genome chloroplast data from 34 *Vitis* genera and found results consistent with the traditional classification.

In this study, an Illumina HiSeq sequencing platform was used to obtain the whole chloroplast genome sequences of four species in the genus *Swertia* L: *S*. *tetraptera*, *S*. *franchetian*, *S*. *przewalskii*, and *S*. *bifolia.* Based on the statistics listed in the National Center for Biotechnology Information (NCBI) database, we found that the chloroplast genomes of 23 species in *Swertia* L., including the four used in this study, have been published. However, most studies on *Swertia* L. have been limited to the publication of single chloroplast genomes, and there have been no systematic analyses of gene structure variations and phylogenetic relationships. Therefore, to obtain a comprehensive and deep understanding of the evolutionary relationships of *Swertia* L. species, all 23 chloroplast genomes were used in this study. The main scientific questions addressed in this study are as follows: 1) How are chloroplast genomes structured and how do they vary across species of *Swertia* L.? 2) What is the phylogenetic relationship between species of *Swertia* L.?

## Materials and methods

### Plant materials

In total, 23 species of *Swertia* L. were selected, four of which were sequenced using Illumina sequencing; the remaining 19 sequences were obtained from GenBank. Fresh young leaves of *S. tetraptera*, *S. franchetian*, *S. przewalskii*, and *S. bifolia* were sampled from Mengyuan county (101.32′ E, 37.62′N, 3,208 m), Huangzhong county (101.63′ E, 36.57′N, 2,510 m), Qilian county (99.61′E, 38.83′N, 3,234 m), and Qilian county (102.22′E, 37.45′N, 3,135 m), respectively, all in the Qinghai province of China. Voucher specimens were deposited in the QTPMB (Qinghai–Tibetan Plateau Museum of Biology) with the voucher numbers QHGC-2011, QHGC20190821, QHGC-2013, and QHGC-2014, respectively. The leaves were dried and preserved in a silica gel.

### Genomic DNA extraction and sequencing

The improved cetyltrimethylammonium bromide method was used to extract the total DNA of *Swertia* L. plants (Doyle, 1991). Agarose gel electrophoresis and a NanoDrop 2000 microspectrophotometer were used to measure the purity and concentration of the DNA. After Illumina PE library was constructed, high-throughput sequencing was completed by Beijing Biomarker Technologies Co., Ltd. Moreover, 150bp paired-end sequencing was performed using Illumina HiSeq (TM) 2000. Raw sequencing data were transformed into sequenced reads (raw data) by performing a base calling analysis of the raw image files. Raw reads data obtained by sequencing were filtered using ngSQCToolkit_v2.3.3 software (Patel and Jain, 2012) to remove low-quality regions and obtain clean reads. The results were then stored in the FASTQ format.

### Assembly, annotation, and sequence analyses

Chloroplast genome assembly was performed using the iterative organelle genome assembly pipeline (Bakker et al., 2016). The chloroplast genome of *S. mussotii* (NC_031155) was used as the reference sequence. SPAdes v3.6.1 software was used for *ab novo* splicing under default parameters and to generate a series of contigs (Prjibelski et al., 2020). Contigs larger than 1,000 bp were used for chloroplast genome assembly. Complete chloroplast genome sequences were constructed by matching and linking contigs (Kearse et al., 2012) and filling the gaps after assembly using second-generation sequencing technology.

The plastid genome annotator tool was used for the functional annotation of *Swertia* L. chloroplast genomes; the start codon, stop codon, and other problematic sites in the annotation result were adjusted manually (Qu et al., 2019; Tian et al., 2021). The annotated chloroplast genome data were exported in Gb format, and the chloroplast genome maps of the four *Swertia* L. species were drawn using OGDRAW (Marc et al., 2013) software. The sequence data and gene annotation information were then uploaded to the NCBI database. The GenBank accession numbers were NC_056357 (*S.franchetiana*), ON164641 (*S.tetraptera*), ON017794 (*S.przewalskii*), and ON018645 (*S.bifolia*).

We used CodonW1.4.2 software to confirm the relative synonymous codon usage (RSCU) and amino acid usage frequency.

### Genome comparison analysis

The chloroplast DNA rearrangement analyses of the 23 *Swertia* L. species were carried out using Mauve alignment (Darling et al., 2004). To show interspecific variation, after annotating the files using Python 3.10.1, the chloroplast genomes of another 22 species of *Swertia* L. were compared using the online software mVISTA (Frazer et al., 2004) and *S. wolfgangiana* as a reference genome. Variations were detected using the Shuffle-LAGAN model. The percentages of variable characters in the coding and non-coding regions were calculated using the method developed by Zhang et al. (2011). IRscope software (Amiryousefi et al., 2018) was used to visually analyze the contraction and expansion of the four boundaries of the 23 species of *Swertia* L.

### Identification of repeat sequences and simple sequence repeats

The online software REPuter (Kurtz et al., 2001) was used to detect repeats in the chloroplast genome, such as forward (F), reverse (R), complementary (C), and palindromic (P). The minimum repetition was set to 30 bp and minimum repetition sequence length distance to 3. In addition, the online program Tandem Repeats Finder was used to detect tandem repeats (Benson, 1999). MISA software (https://pgrc.ipk-gatersleben.de/misa/) was used to predict simple sequence repeat (SSR) in chloroplast genome, and the parameters were set as follows: mononucleotide unit repetition number ≥10; dinucleotide unit repetition number ≥5; trinucleotide unit repetition number ≥4; and tetraconucleotide, pentanucleotide, and hexanucleotide unit repetition number ≥3 (Beier et al., 2017).

### Phylogenetic analysis

In this study, 23 species were used to construct a phylogenetic tree based on Bayesian inference (BI) (Ronquist and Huelsenbeck, 2003), using *Gentianopsis paludosa* (NC_050656) as the outgroup. Mafft v7.205 software was used to compare the sequences and remove irregular sequences at both ends (Kazutaka and Standley, 2013). Before building the BI tree, PAUP and MrModeltest were jointly run through MrMTgui. The Akaike information criterion results showed that the best model for BI analysis was GTR + I + G, with a random tree as the starting tree. Starting with four Markov chains, that is, three hot chains and one cold chain, we saved one tree every 100 generations, calculated 9,000,000 generations, discarded the first 25% preheated (Burn-in) trees, and used the remaining trees to calculate the Bayesian posterior probability (PP) of the consistent tree and each branch.

### Estimation of the divergence times of *Swertia* L. Species

Based on the obtained chloroplast genome sequences, the divergence times of *Swertia* L. species was estimated using the Markov Monte Carlo algorithm (MCMC) molecular sequence Bayesian analysis in BEAST V1.7 (Drummond et al., 2012). First, BEAUti in the software package of BEAST was used to set the parameters of the sequence file in the Nexus format, and the optimal nucleotide substitution model was GTR + I + G, which was selected by MrModeltest. The uncorrelated relaxed clock method was used for the branch lengths with a Gama distribution. Due to the lack of fossil evidence for *Swertia* L. plants, the time was set at 15 Ma (million years), which was from the published literature (Chassot et al., 2001; Von Hagen and Kadereit, 2002; Cao et al., 2021), and the standard variance was 1.0. After a burn-in of 10,000,000 steps, all of the parameters were collected once every 1,000 steps up to 1,00,000,000 Markov chain Monte Carlo (MCMC) algorithm steps. Then an XML format file was generated. The XML format file was imported to BEAST software. The convergence of the MCMC results was detected by using the Tracer v 1.5 program to check that the chain was balanced; we then used the Tree Annotator v 1.7.5 program to obtain the best tree merging and Figtree v 1.4.4 (Rambaut, 2018) was used to view the resulting tree.

## Results and discussion

### Comparison of the chloroplast genomes of 23 *Swertia* L. Species

**FIGURE 1:**
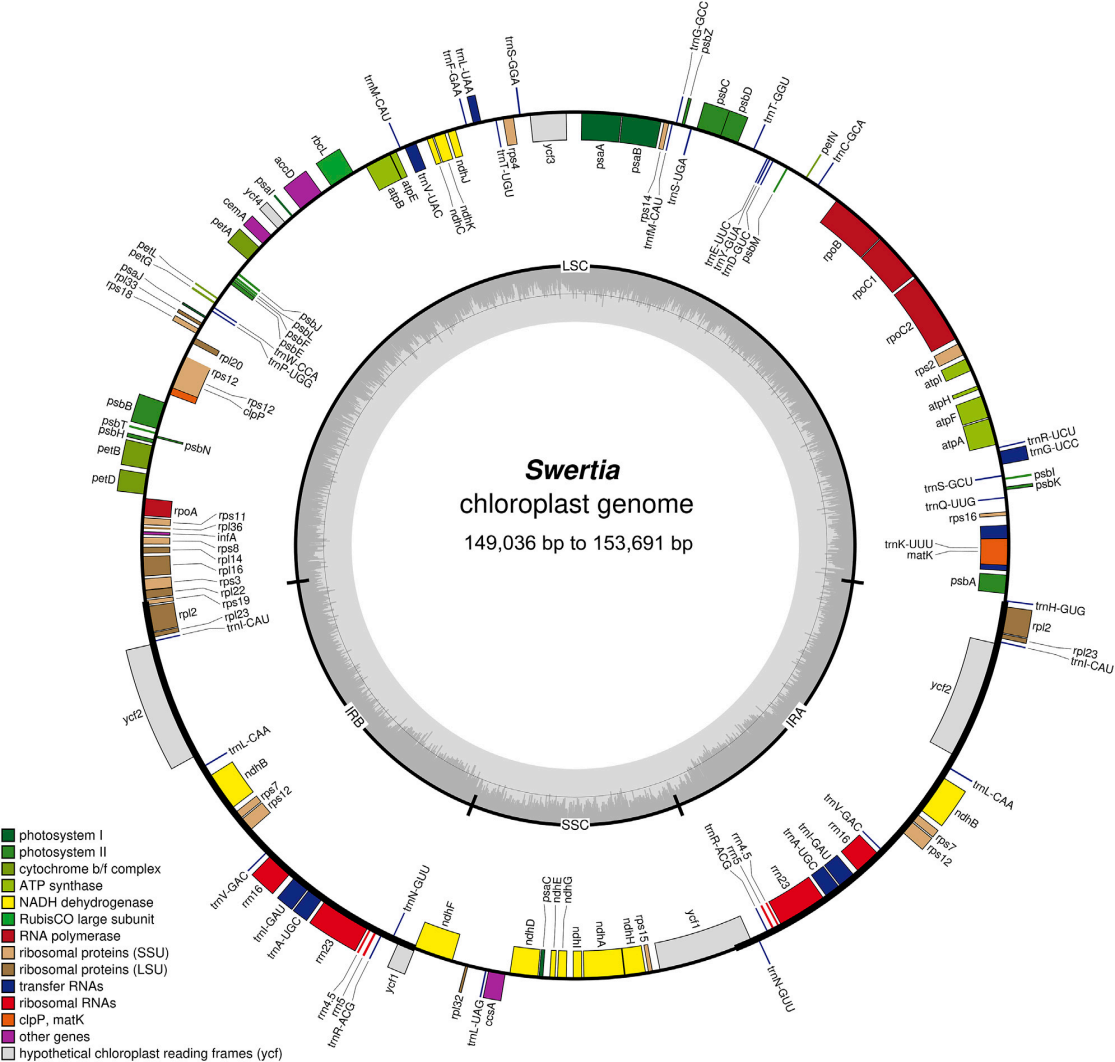
Structure and characteristics of the complete chloroplast genomes of 23 *Swertia* L. species. Genes inside and outside the circle are transcribed clockwise and counterclockwise separately. Darker and lighter grey in the inner circle each represent GC and AT content.

The chloroplast genome lengths of the *Swertia* L. species ranged from 149,036 bp to 153,691 bp, with an average length of 152,385 bp (Table 1). *S. bimaculata* had the longest chloroplast genome, differing from other species in *Swertia* L by 0.06–4.715 kb. As can be seen from the comparison of chloroplast sections, such differences mainly occurred in the large single-copy (LSC) and IR regions. The chloroplast genome length of angiosperms is generally 115–165 kb and that of Gentianaceae is 137–154 kb, which is consistent with the length characteristics of angiosperms and Gentianaceae (Li et al., 2018; Dong et al., 2020). Compared with other genera of Gentianaceae, the average chloroplast genome length of *Swertia* L. was similar to that of *Halenia* (153 kb), but shorter than that of *Paedera* (154 kb) (Dong et al., 2020). The chloroplast genomes of the *Swertia* L. species contained two reverse repeats, IRa and IRb, which divided the whole genome into four parts; the remainder comprised LSC and small single-copy (SSC) regions (Figure 1). The chloroplast genomes of the *Swertia* L. species had the ring tetrad structure typical of angiosperm chloroplast genomes (Palmer, 1985), which made the chloroplast genome highly conserved. The lengths of the LSC regions varied from 80,432 bp to 84,156 bp, with a total GC content of 32.18%–36.35%. The GC content of the SSC region was 31.25%–33.66%, and the total length ranged from 17,887 bp to 18,395 bp. The pair of IRs had a length range of 25,069–25,890 bp and GC content of 42.16%–44.38% (Table 1).

**TABLE 1:** Complete genome features of *Swertia* L. species.

Species	All length (bp)	GC (%)	LSC length (bp)	GC (%)	SSC length (bp)	GC (%)	IR length (bp)	GC (%)	GenBank accession numbers
*Swertia bifolia*	153,242	38.06	83,496	36.16	18,200	31.89	25,773	43.33	ON018645
*Swertia bimaculata*	153,751	38.03	84,156	36.02	18,089	32.07	25,753	43.39	MW344296
*Swertia cincta*	149,089	38.20	80,481	36.34	17,946	31.79	25,331	43.42	MZ261898
*Swertia cordata*	153,429	38.05	83,612	36.16	18,037	31.75	25,890	43.3	NC_054359
*Swertia dichotoma*	152,977	37.5	83,622	35.55	18,092	31.25	25,069	43.02	MZ261899.1
*Swertia dilatata*	150,057	38.17	81,310	36.28	17,887	31.79	25,430	43.42	MW344298
*Swertia diluta*	153,691	38.10	83,859	36.20	18,300	31.9	25,766	43.5	NC057681.1
*Swertia erythrosticta*	153,039	38.10	83,372	36.18	18,249	31.89	25,709	43.33	MW344299
*Swertia franchetiana*	153,428	38.2	83,564	34.66	18,342	33.22	25, 749	43.28	NC_056357
*Swertia hispidicalyx*	149,488	38.19	80,727	36.30	17,903	31.81	25,429	43.42	NC_044474
*Swertia kouitchensis*	153,475	38.15	83,595	36.23	18,348	31.93	25,766	43.47	MZ261902
*Swertia leducii*	153,015	38.17	83,048	36.35	18,395	31.90	25,785	43.44	NC_045301
*Swertia macrosperma*	152,737	38.22	83,046	36.31	18,231	31.99	25,730	43.50	MZ261903
*Swertia multicaulis*	152,190	38.10	82,893	36.25	18,343	31.82	25,477	43.35	NC_050660
*Swertia mussotii*	153,499	38.16	83,591	36.23	18,336	31.95	25,761	43.50	KU641021
*Swertia nervosa*	153,690	38.12	83,864	36.25	18,254	31.82	25,786	43.37	NC_057596
*Swertia przewalskii*	151,079	38.1	81,780	33.22	18,193	33.66	25,553	42.16	ON017794
*Swertia pubescens*	149,036	38.19	80,432	36.33	17,936	31.81	25,334	43.42	MZ261905
*Swertia punicea*	153,448	38.15	83,535	36.25	18,345	31.88	25,784	43.47	MZ261896
*Swertia souliei*	152,804	38.08	83,195	36.17	18,105	31.89	25,752	43.33	NC_052874
*Swertia tetraptera*	152,787	38.1	83,177	32.18	18,305	32.18	25,679	44.38	ON164641
*Swertia verticillifolia*	151,682	38.14	82,623	36.26	18,335	31.83	25,362	43.48	MF795137
*Swertia wolfgangiana*	153,225	38.06	83,528	36.17	18,219	31.88	25,739	43.34	MW344307

Similar chloroplast DNA GC compositions were found in all of the *Swertia* L. species (Table 1), demonstrating high species similarity. The IR regions had a higher GC content than the LSC and SSC regions; this has also been reported in other plants (Choi and Park, 2015; Guo et al., 2020). The IR region contained rRNA and tRNA genes, which accounted for the high DNA GC content of this region (Doorduin et al., 2011; Asaf et al., 2017; Shen et al., 2017).

Most of the chloroplast genomes of angiosperms encode 74 proteins, but some genes have been captured, rearranged, and lost across different families, genera, and species (Millen et al., 2001; Kim et al., 2009). The results of our study showed that *S. bimaculata*, *S. cordata*, *S. diluta*, *S. erythrosticta*, *S. franchetian*, *S. kouitchensis*, *S. leducii*, *S. macrosperma*, *S. mussotii*, *S. punicea*, *S. souliei*, *S. vertickllifolia*, and *S. wolfgangiana* had 133 genes comprising 87 protein-coding genes, 38 tRNA genes, and eight rRNA genes. *S. cincta*, *S. dichotoma*, *S. nervosa*, and *S. pubescens* lacked the *rps16* gene found in the chloroplast genomes of other species of *Swertia* L. Thus, these four chloroplast genomes consisted of 132 genes. The *ycf15* gene in the two reverse repeats was lost in *S. przewalskii* and *S. bifolia*, implying that their chloroplast genomes contained 131 genes. Our result was different from the previous result obtained for Gentianaceae (Dong et al., 2020), which showed that the chloroplast genome of Gentianaceae had 67–80 protein-coding genes, 30 tRNA genes, and four rRNA genes. This difference mainly arose due to gene deletion between genera. For example, the loss of *ndh* genes, including *ndhA*, *ndhC*, *ndhG*, *ndhH*, *ndhI*, *ndhJ*, and *ndhK*, was common to all Gentianaceae species. In addition, four pseudogenes (*ψrps16*, *ψrps19*, *ψinfA*, and *ψycf1*) were present in the chloroplast genomes of the *Swertia* L. species. Previous studies have shown that Gentianaceae plants generally have the same four pseudogenes; our results confirm these previous observations. The *ψinfA* pseudogene likely appeared due to transfer or loss during species evolution (Millen et al., 2001; Zhou et al., 2016). The appearance of the *ψrps19* and *ψycf1* pseudogenes is likely due to their location at the boundary of the chloroplast gene region, which experiences a boundary effect (Li et al., 2018). The second missing exon in the *ψrps16* pseudogene was first detected in *Gentiana macrophyllum* (Ni et al., 2016) and in non-parasitic species of the *Chrysanthemum* branch (APG IV). Since then, the *ψrps16* pseudogene has been detected in the chloroplast genomes of several Gentianaceae members, the structures of which are similar across all species.

The functions of the major genes in the chloroplast genomes of *Swertia* L. were roughly classified into three categories (Table 2): chloroplast self-replication–related genes, photosynthesis-related genes, and other genes (Saski et al., 2005). Genes related to photosynthesis and self-replication accounted for the majority of the chloroplast genome.

**TABLE 2:** Gene composition of chloroplast genome of all *Swertia* L. species.

Category	Group of genes	Name of genes
Photosynthesis	Photosystem I	*psa*A, *psa*B, *psa*C, *psa*I, and *psa*J
	Photosystem II	*psb*A, *psb*B, *psb*C, *psb*D, *psb*E, *psb*F, *psb*H, *psb*I, *psb*J, *psb*K, *psb*L, *psb*M,*psb*N, *psb*T, and *psb*Z
	NADH dehydrogenase	*ndh*A^a^, *ndh*B^a^, *ndh*C, *ndh*D, *ndh*E, *ndh*F, *ndh*G, *ndh*H, *ndh*I, *ndh*J, and *ndh*K
	Cytochrome b/f complex	*pet*A, *pe*tB, *pet*D, *pet*G, *pet*L, and *pet*N
	ATP synthase	*atp*A, *atp*B, *atp*E, *atp*F^a^, *atp*H, and *atp*I
Self-replication	Ribosomal proteins (SSU)	*rps*2, *rps*3, *rps*4, *rps*7, *rps*8, *rps*11,*rps*12^c^, *rps*14, *rps*15,*rps*16, *rps*18, and *rps*19
	Ribosomal proteins (LSU)	*rpl*2^a^, *rpl*14, *rpl*16, *rpl*20, *rpl*22, *rpl*23, *rpl*32, *rpl*33, and *rpl*36
	Ribosomal RNAs	*rrn*4.5^1^, *rrn*5^1^, *rrn*16^1^, and *rrn*23^1^
	Transfer RNAs	tRNA-Lys^a^, tRNA-Gln, tRNA-Ser, tRNA-Gly^a^, tRNA-Arg, tRNA-Cys, tRNA-Asp, tRNA-Tyr, tRNA-Glu, tRNA-Thr, tRNA-Ser, tRNA-Gly, tRNA-Met, tRNA-Ser, tRNA-Thr, tRNA-Leu, tRNA-Phe, tRNA-Val, tRNA-Gly, tRNA-Met, tRNA-Trp, tRNA-Pro, tRNA-Ile, tRNA-Leu^a^, tRNA-Val^a^, tRNA-His, tRNA-Ile^a^ ^1^, tRNA-Ala^a^1, tRNA-Arg1, tRNA-Asn1, tRNA-Leu, tRNA-Asn, tRNA-Arg, tRNA-Ala, tRNA-Ile, and tRNA-His
	DNA-dependent RNA polymerase	*rpo*A, *rpo*B, *rpo*C1^a^, and *rpo*C2
Other genes	Maturase	matK
	Protease	clpP^a^
	Envelope membrane protein	cemA
	Subunit acetyl-CoA carboxylase	Accd
	c-Type cytochrome synthesis gene	ccsA
Genes of unkown function	Conserved open reading frames	ycf1, 2a, 3^a^, 4, and 15

a represents a gene with one intron.

b represents a gene with two introns.

c represents trans-splice gene.

Further analysis of the chloroplast genes of *Swertia* L. showed that they were similar to those of other plants and that most did not contain introns (Du et al., 2018; Guo et al., 2020). In this study, only 16 genes (*rps12*, *trnK-UUU*, *atpF*, *rpoC1*, *ycf3*, *trnL-UAA*, *trnV-UAC*, *clpP*, *petB*, *petD*, *rpl16*, *rpl2*, *ndhB*, *trnI-GAU*, *trnA-UGC*, and *ndhA*) in the chloroplast genomes of *Swertia* L. contained introns, and all of them contained one intron except for the *clpP* and *ycf3* genes, which had two introns (Table 2). The *rps12* gene in the chloroplast genomes of *Swertia* L. experienced trans-splicing, in which the 3′ end was in the IR region and 5′ end was in the LSC region. This phenomenon has been observed in the majority of other land plants (Du et al., 2018).

The preference of 59 synonymous codons was evaluated using RSCU (Wu et al., 2007). Based on the statistical analysis, the number of codons in the *Swertia* L. species varied from 49,696 to 512,30. Leucine (Leu; 4,988–5,394 codons), isoleucine (Ile; 3,730–4,277 codons), and phenylalanine (Phe; 3,498–3,641 codons) were the three amino acids with the highest coding rates in the *Swertia* L. species chloroplast genomes. Only 663–719 codons encoded tryptophan (Trp), which had the lowest coding rate among all of the amino acids (Supplementary Table S1).

### Repeat sequences and simple sequence repeats

**FIGURE 2:**
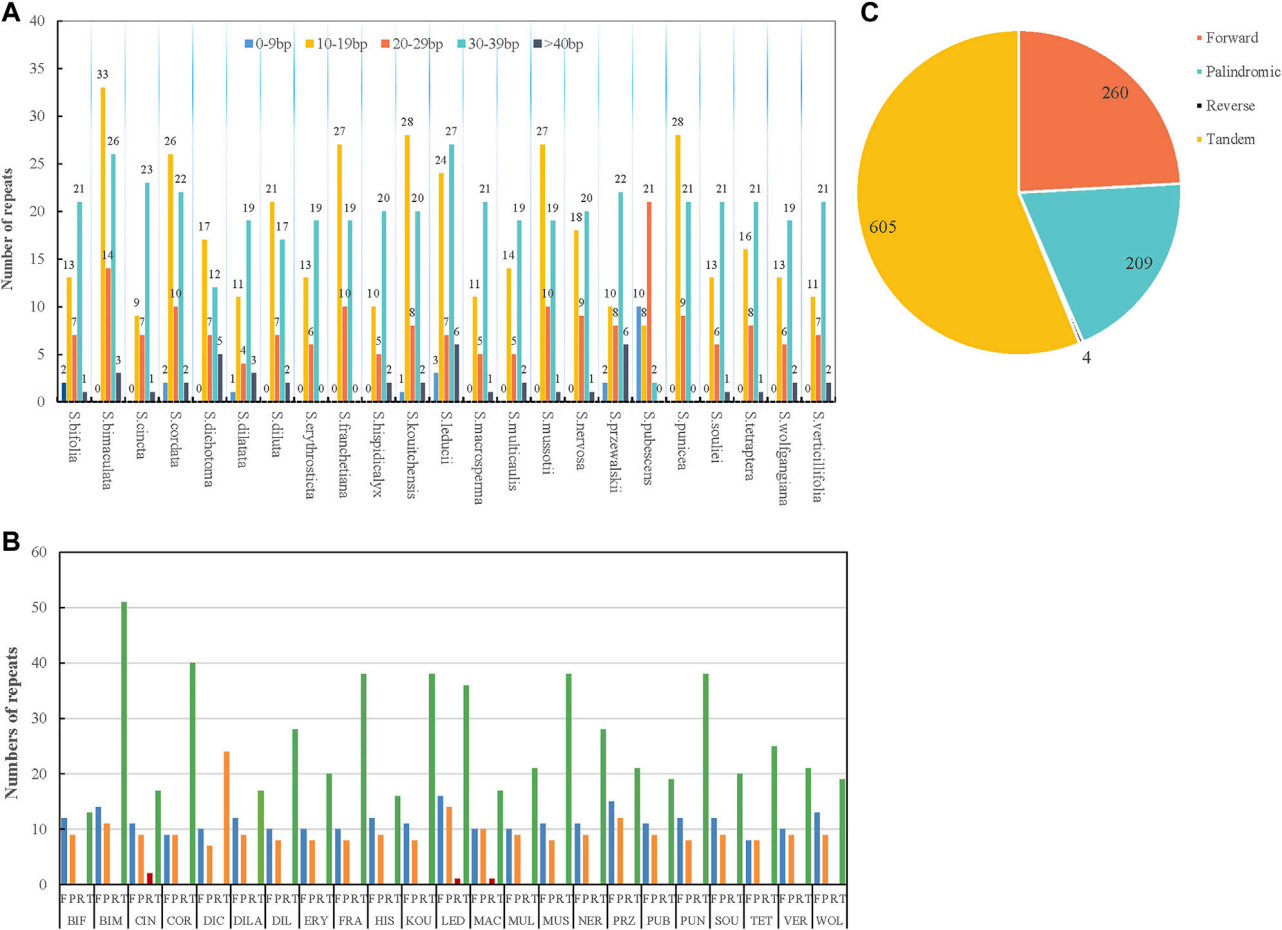
Type of repeated sequences in the 23 *Swertia* L. plastid genomes. **(A)** Number of repeat sequences by length; **(B)** number of four repeat types (Note: BIF represents *S. bifolia*; BIM represents *S. bimaculata*; CIN represents *S. cincta*; COR represents *S. cordata*; DIC represents *S. dichotoma*; DILA represents *S. dilatata*; DIL represents *S. diluta*; ERY represents *S. erythrosticta*; FRA represents *S. franchetiana*; HIS represents *S. hispidicalyx*; KOU represents *S. kouitchensis*; LED represents *S. leducii*; MAC represents *S. macrosperma*; MUL represents *S. multicaulis*; MUS represents *S. mussotii*; NER represents *S. nervosa*; PRZ represents *S. przewalskii*; PUB represents *S. pubescens*; PUN represents *S. punicea*; SOU represents *S. souliei*; TET represents *S. tetraptera*; VET represents *S. verticillifolia*; and WOL represent *S. wolfgangiana*); **(C)** pie chart showing the numbers of four repeat types.

Repetitive sequences are the main sources of duplication, deletion, and rearrangement in the chloroplast genome (Li and Zheng, 2018). In this study, four kinds of repetitions were counted: forward, palindromic, tandem, and reverse. The results showed that the distributions and numbers of repeats in the 23 chloroplast genomes were similar and conserved (Figure 2; Supplementary Table S2). Tandem units were the most repeated type (605), followed by forward (260), palindromic (209), and reverse repeats (4) (Figure 2C). There were interspecific differences in the tandem repeats, but the ratio of forward to palindromic repeats was about 1:1. Reverse repeats only existed in *S. cincta*, *S. leducii*, and *S. macrosperma*. The lengths of the repeat units were mainly 8–39 bp (Figure 2A). The majority of repetitive sequences were scattered across intergenic or intronic regions, with only a few distributed across gene regions such as *ycf3*, *ycf2*, *ndhE*, *psaB*, *accD*, *petB*, *ndhA*, *psbA*, *accD*, *rps18*, *rps16*, *psbK*, *clpP*, *ycf1*, *atpH*, and *rps2* (Supplementary Table S2). *S. bimaculata* had the most repeat sequences (76) of all the analyzed *Swertia* L. species, followed by *S. leducii* (67); *S. bifolia* had the fewest repeat sequences (34) (Figure 2B).

**FIGURE 3:**
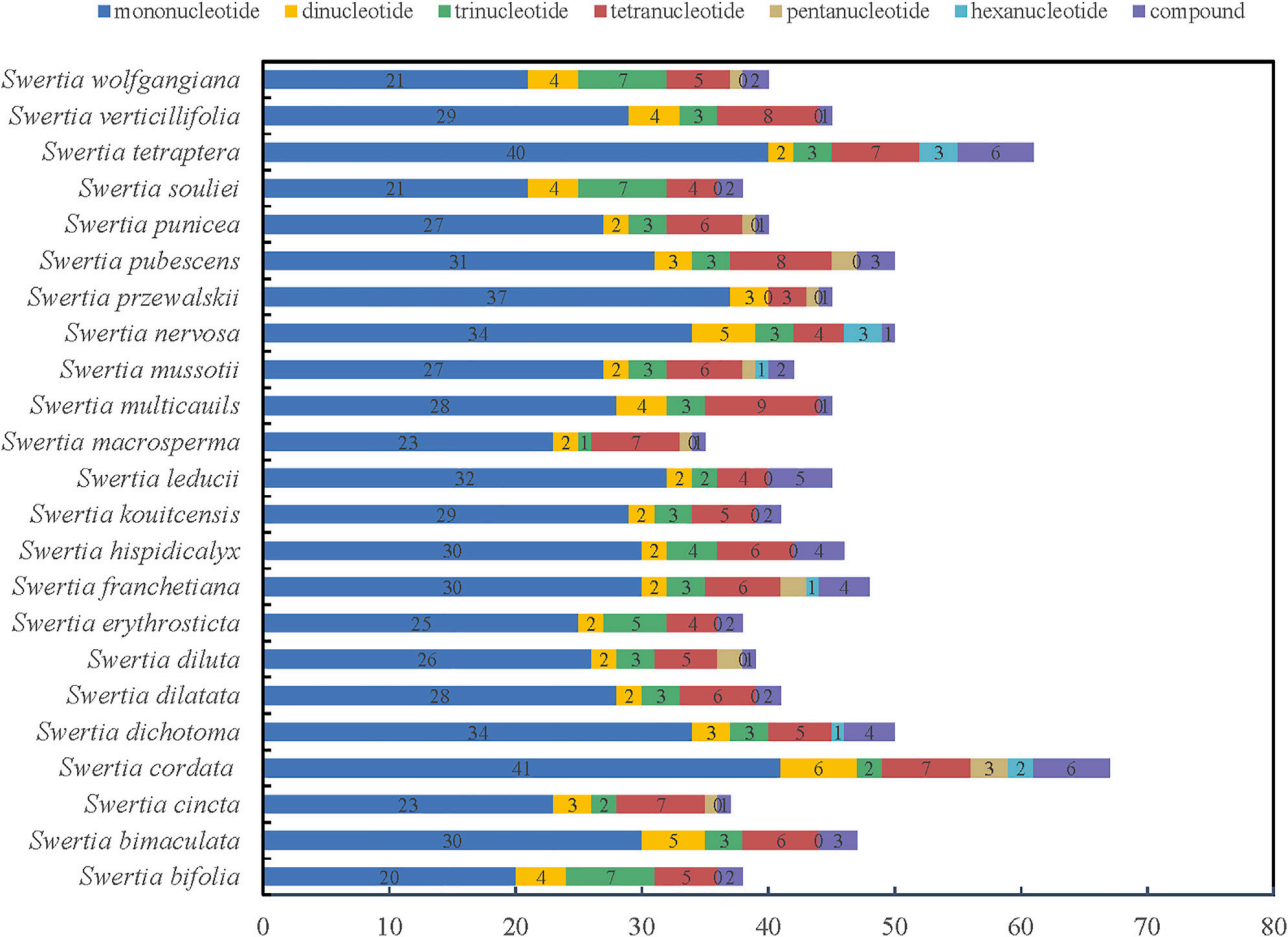
Simple sequence repeats (SSRs) in the 23 *Swertia* L. plastid genomes.

As a classical molecular marker, simple repeat sequence (SSR) has been widely used in the analysis of population genetic evolution. We analyzed the simple repeat sequence (SSR) in the chloroplast genomes of 23 species of *Swertia* L. and the result showed that the numbers of SSR ranged from 35 to 61. *S. tetraptera* had the most SSRs (61) and *S macrosperma* had the fewest SSRs (38). Moreover, the numbers and types of mononucleotide, dinucleotide, trinucleotide, tetranucleotide, pentanucleotide, and hexanucleotide repeats were also different in the 23 species of *Swertia* L. (Figure 3; Supplementary Table S3). Mononucleotides were the most common repeat type. The proportion of mononucleotides in all SSRs ranged from 50.00% to 82.22% in 23 species of *Swertia* L. This finding is in accordance with the previous observation (Kuang et al., 2011). In total, 70 dinucleotides were detected in 23 species, which were AT/TA, accounting for 3.23%–10.53% of the SSRs. In total, 76 trinucleotides and 133 tetranucleotides were found in the 23 complete cp genomes. A total of 15 pentanucleotides were discovered in chloroplast genes of 23 species in *Swertia* L. Only S. cordata (2), *S. dichotoma* (1), *S. franchetiana* (1), *S. mussotii* (1)*, S. nervosa* (3), and *S. tetraptera* (3) had hexanucleotides. In addition, compound SSRs accounted for 2.17%–10.87% of the 23 genomes. The richness of SSRs and the count of SSRs were different within *Swertia* L. thus these may be helpful molecular marker for species identification. However, adopting SSRs to clarify ecological and evolutionary processes has yet to be fully implemented (Ebert and Peakal, 2009). The results of this study will provide a basis for the study of chloroplast SSR markers in the future and lay a foundation for the study of the genetic relationship and diversity of this genus.

Oligonucleotide repeats are widely found in the plastome (Ahmed et al., 2012, 2013; Abdullah et al., 2021). These repeats have an effect on generating mutations and have been suggested as a proxy for mutational hotspots (Ahmed et al., 2012; Ahmed et al., 2012; Abdullah et al., 2020; Abdullah et al., 2021). Abdullah et al. (2020) proposed that the co-occurrence of repeats with substitutions was up to 90%, whereas 36%–91% co-occurrence was found at the genus level. In the present study, 10 highly polymorphic loci were found. Among these, five loci belong to the regions where repeats are present, including *psaA*-*ycf3* and *rps15*, which showed the highest incidence of polymorphisms. Here, our findings support the use of repeats as a proxy, and this approach may also be helpful for the identification of suitable polymorphic loci for phylogenetic inference of other taxonomically complex genera. This approach is promising since the plastome of a single species can be used to identify polymorphic regions. Repeated coding regions and IR regions need to be avoided, however, due to the purifying selection pressure of protein-coding genes (Henriquez et al., 2020) and the fact that copy-dependent repair mechanisms (Zhu et al., 2016) lead to low rates of mutation.

### Sequence divergence across *Swertia* L. species

**FIGURE 4:**
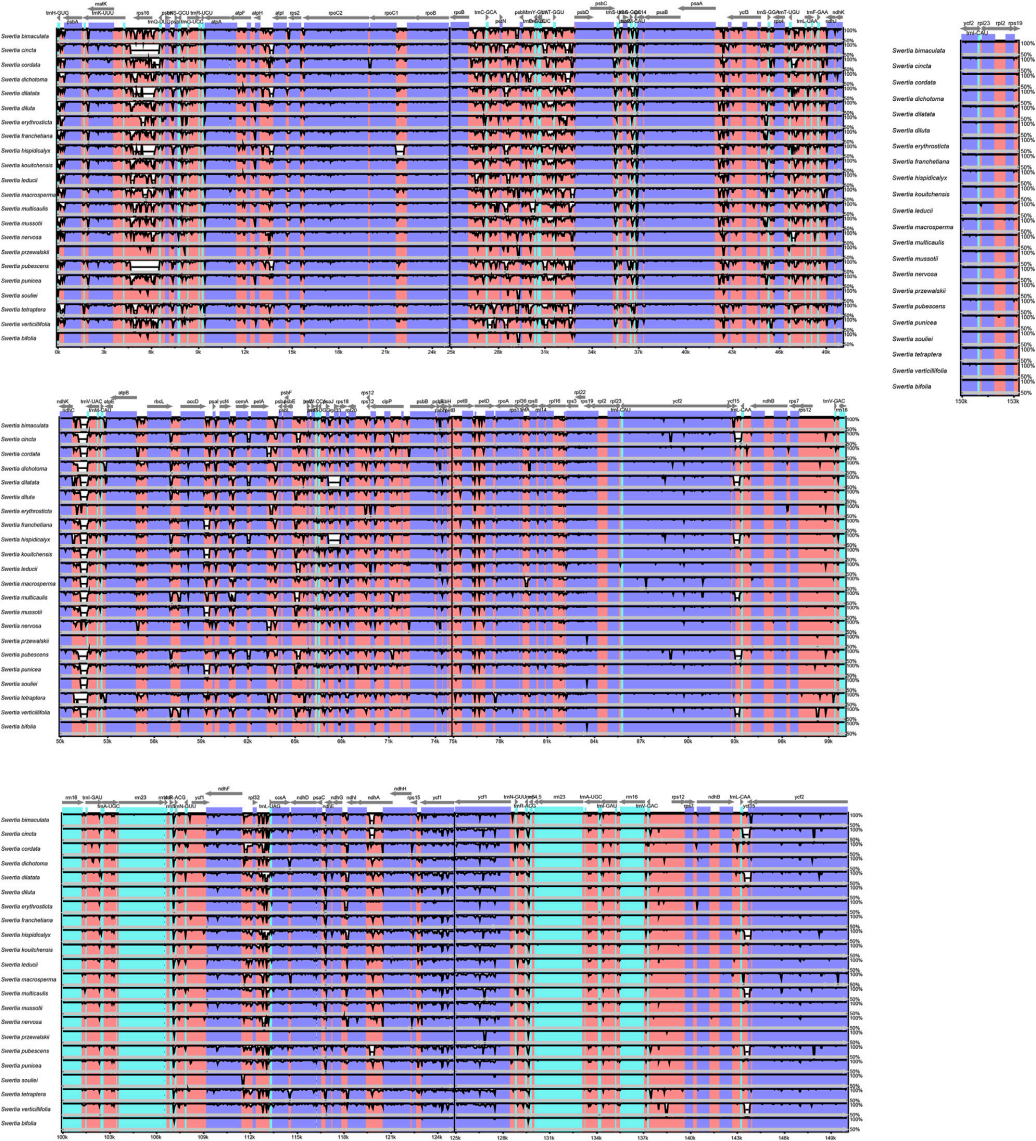
Comparison and analysis based on chloroplast genome of 23 *Swertia* L. species. Orientation of genes was pointed out by arrows up the alignments. Purple, blue, pink, and grey bars correspond to exons, untranslated regions, non-coding sequences, and mRNA, respectively. The *Y*-axis indicates the genetic similarity percentage. Genetic similarity among 50%–100% were showed in the figure (for interpretation of the references to color in this figure legend, the reader is referred to the web version of this article).

**FIGURE 5:**
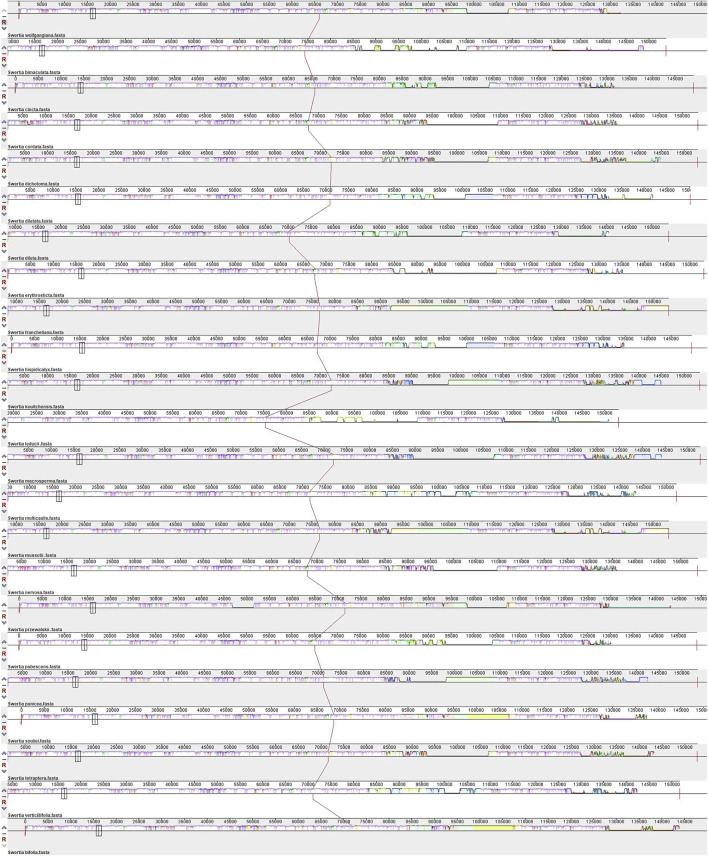
MAUVE alignment of 23 *Swertia* L. species chloroplast genomes. The *S* ***.*** *wolfgangiana* genome is shown at the top as the reference genome.

**FIGURE 6:**
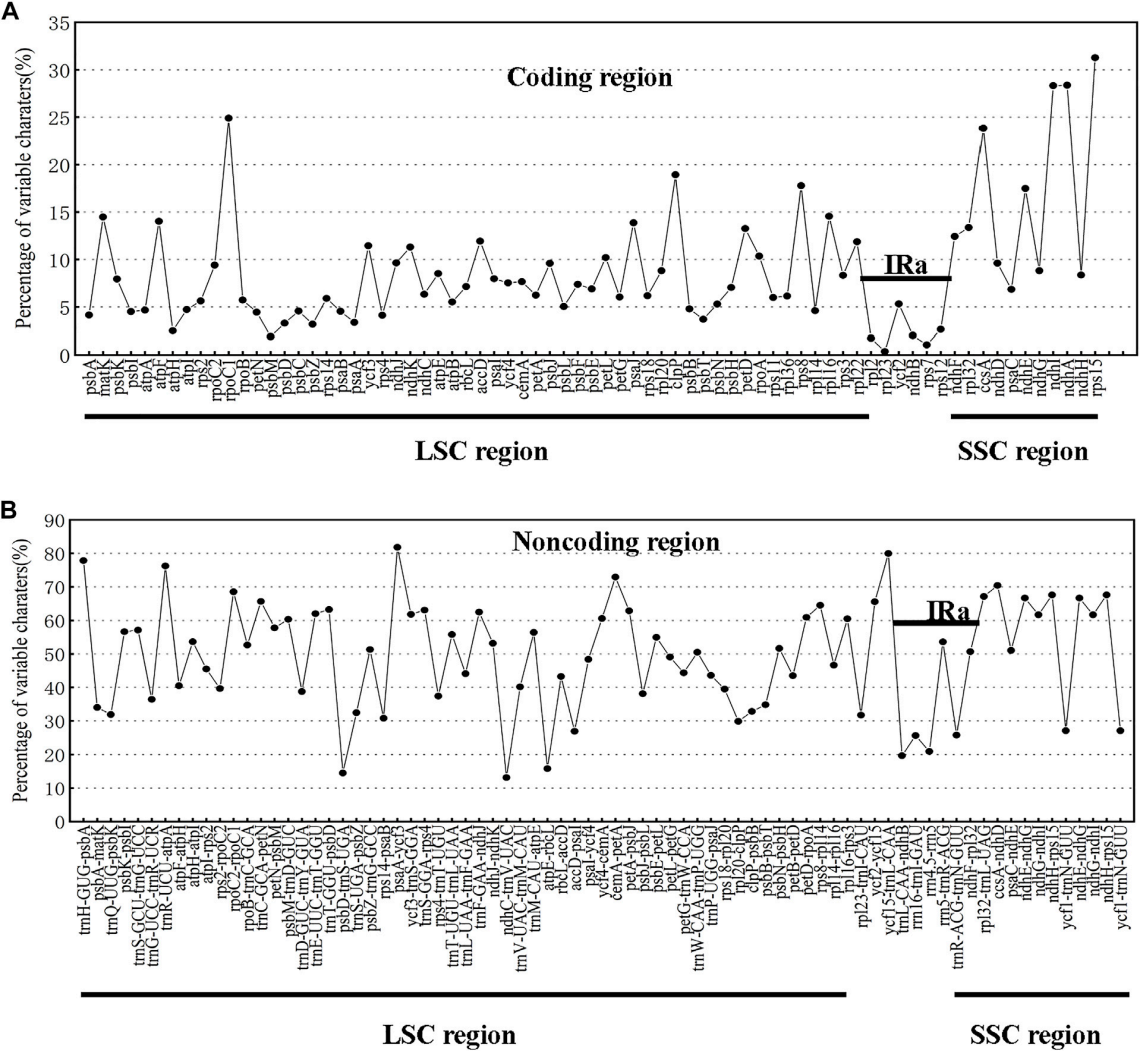
Percentages of variable characters in homologous regions among chloroplast genomes of 23 *Swertia* L. species. **(A)** Coding region. **(B)** Non-coding region. The homologous regions are oriented according to their locations in the chloroplast genome.

The chloroplast genomes of the 23 *Swertia* L. species were relatively conserved, with four parts of the genomes being arranged in consistent sequences (Figure 4) and no rearrangement found in gene organization after verification (Figure 5). Moreover, there was a higher degree of variation in non-coding regions than in the coding regions of the chloroplast genome of *Swertia* L. In the non-coding regions, the percentage of variations ranged from 13.14% to 81.84% (Figure 6B), averaging 49.02%, whereas in coding regions, the percentage of variations ranged from 0.35% to 31.27%, averaging 9.10% (Figure 6A). The SSC region variability of the 23 species in *Swertia* L. was higher than that of the LSC and IR regions in both coding (7.96%, 2.19%, and 17.16% for LSC, IR, and SSC regions, respectively) and non-coding regions (49.00%, 42.44%, and 54.23% for LSC, IR, and SSC regions, respectively). The degree of variation was lowest in the IR region, indicating a high degree of conservatism. These results were consistent with those obtained for other angiosperms (Dong et al., 2013; Guo et al., 2020). In addition, some genes (*rpoC1*, *ccsA*, *ndhI*, *ndhA*, and *rps15*) exhibited higher variability than other genes in the 23 species of *Swertia* L. Some of the non-coding regions with high sequence divergence were *trnH-GUG*-*psbA*, *psaA*-*ycf3*, *cemA*-*petA*, *ycf15*-*trnL-CAA*, and *ccsA*-*ndhD*. These genes and hotspot regions can either be used in phylogenetic analyses or serve as potential DNA molecular barcodes (Zhang et al., 2011; Maier et al., 1995; Diekmann et al., 2009).

### IR contraction and expansion of the chloroplast genome

**FIGURE 7:**
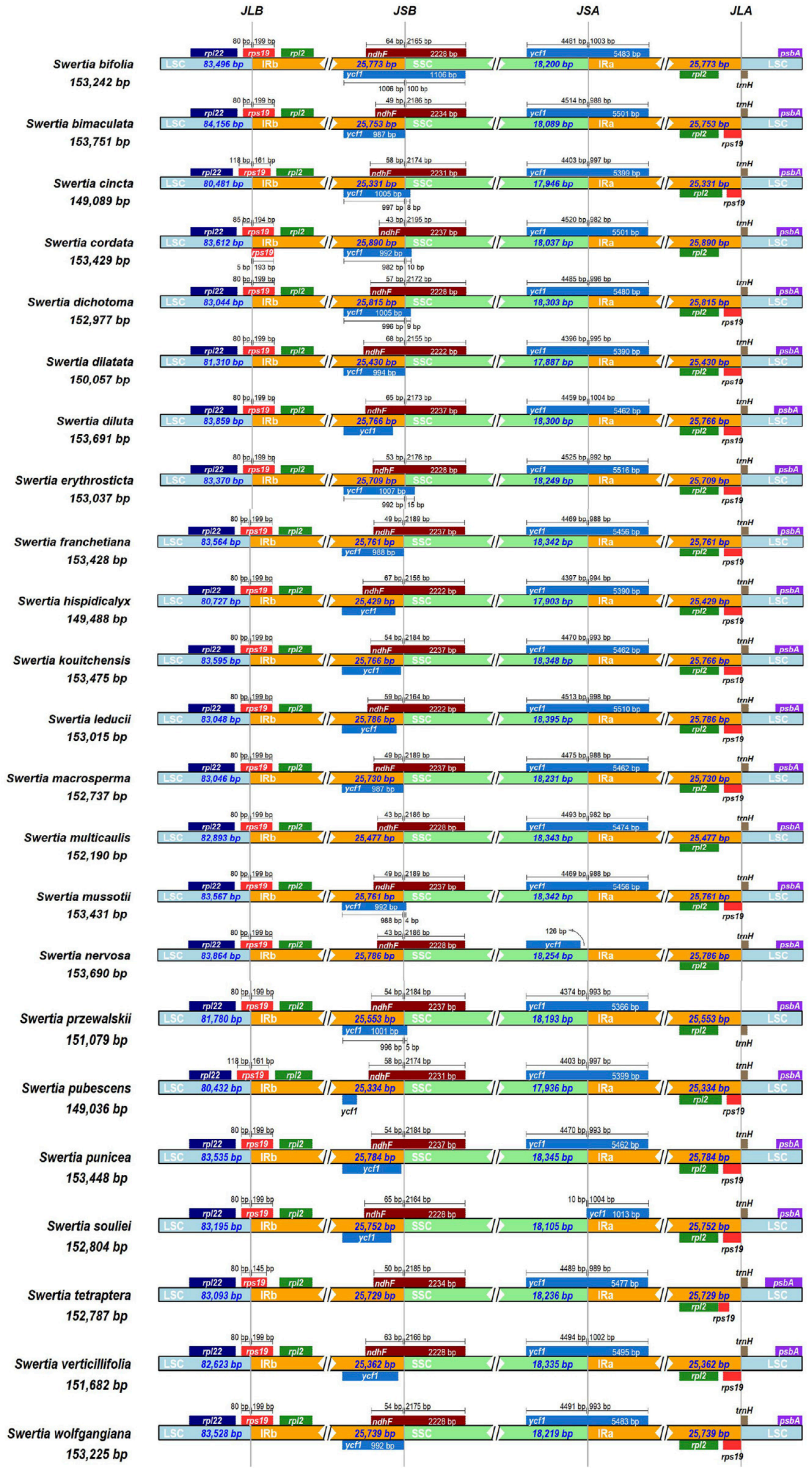
Comparative analysis of chloroplast genomic boundaries of the 23 *Swertia* L. plastid genomes.

The chloroplast genome has two IR regions, which form four boundaries with LSC and SSC regions: IRb-LSC, IRb-SSC, IRa-LSC, and IRa-SSC. When the ancient genome evolved, the IR boundary expanded and contracted, causing some genes to enter IR regions and some to enter the single-copy regions, with different levels of sequence replication at each species boundary. As can be seen from Figure 7, the four boundaries of the chloroplast genomes of the *Swertia* L. species were relatively well-conserved. The *rps19* gene spanning the LSC and IRb regions was present at the IRb-LSC boundary in all 23 *Swertia* L. chloroplast genomes. This gene was mainly located in the LSC region at the same bases, except in *S. cordata* (85), *S. cinata* (118), and *S. pubescens* (118). The IRa-LSC boundaries in most of the *Swertia* L. chloroplast genomes occurred between the *rps19* pseudogene in the IRa region and the *trnH* gene in the LSC region; however, the *rps19* pseudogene was absent in *S. bifolia*, *S. przewalskii*, *S. nervosa*, and *S. multicaulis*. The IRb-SSC boundaries in the *Swertia* L. chloroplast genomes varied greatly. This boundary was located in the overlapping region of the *ycf1* pseudogene and *ndhF* gene in 11 *Swertia* L. chloroplast genomes, with the IRb-SSC boundary in six *Swertia L.* chloroplast genomes crossing the overlap region and extending 5–100 bp to the *ndhF* gene. The *ycf1* pseudogene in eight *Swertia* L. chloroplast genomes was present in the IRb region, along with a terminal from the IRa-SSC border. In addition, the *ycf1* pseudogene was lost in the IRb-SSC boundaries of the chloroplast genomes in *S. tetraptera*, *S. nervosa*, and *S. multicaulis* (Figure 6). The IRa-SSC boundary was located in the *ycf1* gene in all of the species, but the length of the *ycf1* gene fragment in the IRa region differed to some extent and ranged from 988 bp to 1,004 bp. The length of this fragment was about 5,400 bp in most *Swertia* L. species, except for *S. nervosa* and *S. souliei*. The *ycf1* gene in the *S. nervosa* chloroplast genome was present in the SSC region, with a terminal 126 bp from the IRa-SSC border. The total length of the *ycf1* gene in the *S. souliei* chloroplast genome was 1,013 bp, with only 10 bp located in the SSC region. The sliding of the IRa-SSC and IRb-LSC boundaries in the chloroplast genomes of vascular plants generally occurs in different genera or even within the same genus, resulting in large variations in chloroplast genome length across different plants. The IRb-LSC boundaries of the *Swertia* L. species were largely located within the *rps19* gene and, as mentioned earlier, the IRa-LSC boundary was located between the *rps19* gene of the IRa region and the *trnH* gene of the LSC region. However, in monocotyledon plants such as those in the Orchidaceae and Poaceae families, the boundaries are extended and the *rps19* and *trnH* genes are located in the IR regions (Tang et al., 2011; Hu, 2020). Both genes changed from one to two copies, whereas in barley and sorghum, boundary shrinkage occurred, resulting in two copies of the *rps19* and *trnH* gene in the LSC region (Tang et al., 2011). The IRb-SSC boundary was located in the *ycf1-ndhF* overlap region in 11 *Swertia* L. species, which is consistent with the observations from many species of cruciferous plants (Li et al., 2017), for example, *Aethionema grandiflorum*, *Arabidopsis thaliana*, *Barbarea verna*, *Brassica napus*, *Cakile arabica* and so on. The extension of the IRb-SSC boundary to the *ndhF* gene due to boundary expansion has also been detected in *Arabidopsis thaliana* (Tang et al., 2011), in which the IRa-SSC boundary is located in the *ycf1* gene. In *A. thaliana*, the fragment lengths of the *ycf1* gene in the SSC and IRa regions are different due to either contraction or expansion of the boundary. In rice, wheat, maize, and other plants, this boundary is located on the *ndhH* gene, further indicating that the boundary between dicotyledons and monocotyledons is quite different (Melodelima et al., 2013).

People have different views on the mechanism of contraction and expansion of the IR region (Guo et al., 2020). DNA double-strand breaks (DSBs) are currently considered the main molecular mechanism underlying IR expansion. As large contractions in the IR region are rare, the DSB theory may also underlie IR region contraction.

### Phylogenetic analysis

**FIGURE 8:**
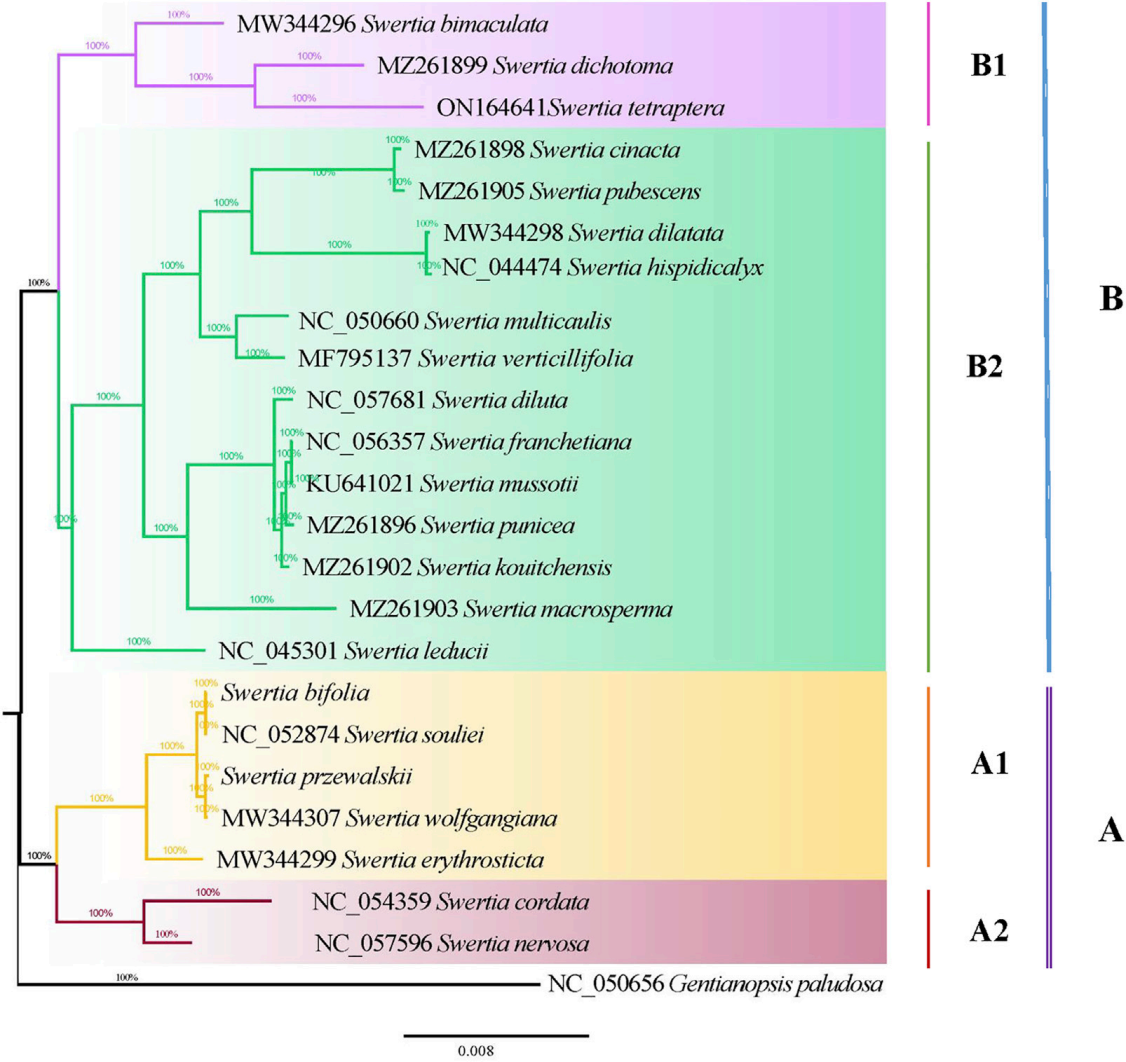
Phylogenetic tree of 23 *Swertia L.* species using Bayesian inference (BI) analyses based on whole chloroplast genomes.

The maximum likelihood and Bayesian methods were used to construct phylogenetic trees for the chloroplast genomes of the 23 *Swertia* L. species. The topological structures of the phylogenetic trees obtained using the two methods were similar (Figure 8). Phylogenetic analysis showed that all 23 species of *Swertia* L. in conjunction with those of *G. paludosa,* formed a well-supported clade, indicating that the genus *Swertia* L. was not monophyletic. This result is supported by previous studies (Chassot et al., 2001; Struwe and Albert, 2002; Von Hagen and Kadereit, 2002; Favre et al., 2010; Xi et al., 2014; Cao et al., 2021). In addition, the well-supported clade was divided into two major clades (A and B), corresponding to the subgen. *Swertia* (A) and subgen. *Ophelia* (B).

Seven species in *Swertia* L. were clustered into a subgen. *Swertia* phylogenetic tree at the base, which showed a close genetic relationship. Ho et al. (1994) believed that this subgenus was a relatively primitive group of *Swertia* L. consisting of a perennial herb with ancestral traits such as a single stem and unbranched large flowers. Within the same clade, the four species of sect. *Swertia* (*S. souliei*, *S. bifolia*, *S. wolfgangiana*, and *S. erythrosticta*) formed a single clade (A1) and two species (*S. cordata* and *S. nervosa*) of sect. *Ophelia* formed another clade (A2). These two were sister branches, further supporting the division of these groups by Ho et al. (1994). Clade B had two branches: B1 and B2 subclades. The B1 subclade contained *S. bimaculata*, which belonged to sect. *Ophelia*. This clade also included an *S. dichotomy*–*S. tetraptera* branch. *S. bimaculata* and *S. dichotomy*–*S. tetraptera* were sisters. The plants in these two branches were closely related (100 bootstrap), indicating a common ancestor. The B2 subclade contained sect. *Ophelia*, sect. *Platynema*, sect. *Poephila*, and sect. *Macranthos*. In this subclade, *S. leducii* was differentiated first and located at the base. Furthermore, two parallel branches were then isolated: sect. *Ophelia* and sect. *Platynema*; sect. *Poephila* and sect. *Macranthos*. *S. multicaulis*, from subgen. *Poephila*, and *S. verticillifolia*, from sect. *Macranthos*, were first clustered into a small clade and then into a large clade with the three species of sect. *Platynema* and one species of sect. *Ophelia*. This differed from the morphological classification. Sect. *Platynema* was at the top of the B2 subclade, indicating that it was located in a comparable evolutionary position of the phylogenetic tree of *Swertia* L. The clustering results partially validated the results obtained by Ho et al. (1994), who showed that sect. *Platynema* and sect. *Kingdon-Wardia* (Marq.) were the most evolved groups of the genus and characterized by extremely enlarged filaments at the base, a single glandula in each corolla lobe, and diminished tassels. In the present study, sect. *Kingdon-Wardia* (Marq.) was not included in the phylogenetic tree, making it impossible to show its systematic position. However, sect. *Platynema* and sect. *Kingdon-Wardia* (Marq.) were clustered together and located in the same relative evolutionary branch of *Swertia* L. in a study by Xi et al. (2014). From what has been discussed before, the division of the two subgenera (subgen. *Swertia* and subgen. *Ophelia*) and five sections (sect. *Ophelia*, sect. *Platynema*, sect. *Poephila,* sect*. Swertia*, and sect. *Macranthos*) is partially supported by molecular data. However, the systematic positions of other sections and species in *Swertia* L. derived from molecular data differed from the morphological classification. Inconsistencies between different data types, specifically between morphological and molecular data, remain a major problem of systematics (Lee, 2001). Such inconsistencies have been reported and discussed for many plant and animal groups, such as Rubiaceae, Loganiaceae, *Isothecium*, and Dendrocolaptinae (Bremer and Struwe, 1992; Irestedt et al., 2004; Draper et al., 2007). Pisani et al. (2007) argued that despite the widespread inconsistencies between morphological and molecular data, both data types were equally important in estimating phylogenetic relationships and that molecular data could not be considered more reliable. The results of this study were roughly equivalent to those of previous studies that used different gene fragments and species to examine the phylogeny of *Swertia* L. indicating a conflict between the morphological classification system and molecular data, which can be explained from the perspective of evolution. The formation of new species is a slow process, usually occurring over thousands of years. Variations due to natural selection and genetic drift become fixed in a group, driving the formation of new species that eventually differ from two recent common ancestors, that is, species derived from two recent common ancestors, both morphologically discontinuous and reproductively isolated, are monophyletic (Liu, 2016). Driven by the drastic changes in the geology and climate of the Qinghai–Tibetan Plateau, the ancestors of *Swertia* L. evolved rapidly and showed abundant morphological diversity, such as in the shape and length of the corolla and number and location of nectaries, nectary appendages, and corolla throat appendages. However, this taxon has not accumulated enough sequence variation for a molecular phylogenetic analysis over a relatively short period of time. Moreover, mutations in gene sequences have not been fixed in the population by genetic drift. In addition, the uniparental inheritance of the plastome may also confound phylogenetic inference. Previous studies have shown that the phylogeny based on plastome and mitochondria sequences contradicted with nuclear due to uniparental inheritance of these genomes (Vargas et al., 2017; Abdullah et al., 2021). Therefore, more genetic markers (nuclear) and more taxa of *Swertia* L will be needed to further explore the phylogenetic relationships in this genus.

### Divergence time of *Swertia* L. Species

**FIGURE 9:**
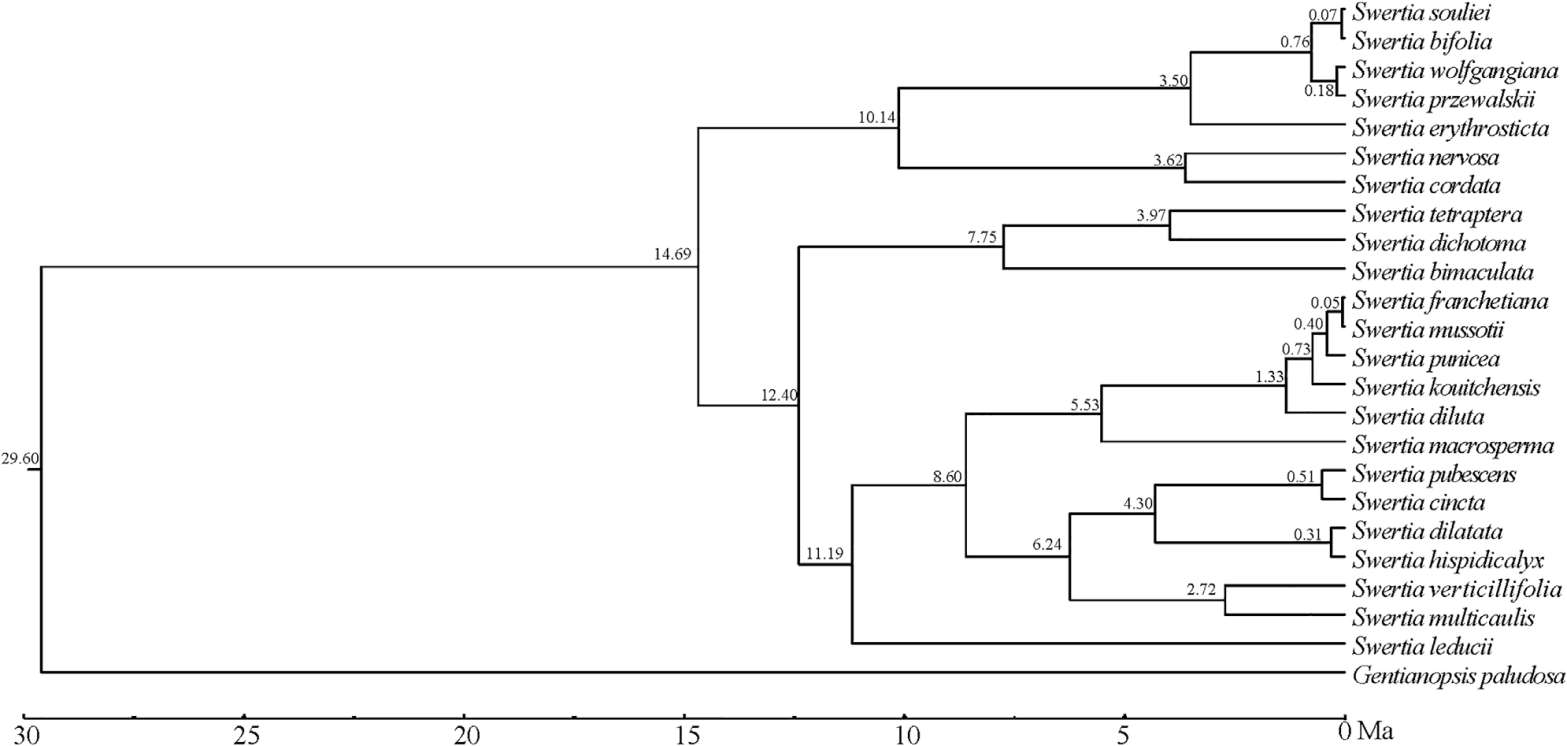
Divergence time estimated using BEAST.

Tracer v 1.5 was used to check the analysis values of each parameter, and it was shown that the number of MCMC iterations calculated by BEAST had met the effective sample size (ESS), which was greater than 200. The BEAST analysis was based on the phylogenetic trees of chloroplast genomes of 23 species of *Swertia* L.(Figure 9), and the numbers at each branch node of the phylogenetic tree were the divergence times (Ma) of the corresponding groups. The result showed that the estimated divergence between *Swertia* L. and *Gentianopsis* occurred at 29.60 Ma. We therefore inferred that *Swertia* L formed at 29.60 Ma, corresponding to the early Miocene of the Tertiary. Meanwhile, the divergence between subgen. *Swertia* and subgen. *Ophelia* appeared at 14.69 Ma. In addition, the estimated divergence time in 23 species of *Swertia* L. was between 12.40 and −0.05 Ma. The formation of *S. franchetiana*, *S. mussotii*, *S. punicea*, *S. kouitchensis*, *S. diluta*, *S. pubescens*, *S. cincta*, *S. dilatata*, *S. hispidicalyx*, *S. souliei*, *S. bifolia*, *S. wolfgangiana*, and *S. przewalskii* were at 0.05–1.33 Ma (the Quaternary), and *S. macrosperma*, *S. erythrosticta*, *S. nervosa*, *S. cordata*, *S. tetraptera*, *S. dichotoma*, *S. bimaculata*, *S. verticillifolia*, *S. multicaulis*, and *S. leducii* were formed at 2.72–12.40 Ma (end of Tertiary).

In the present study, the formation of *Swertia* L. was dated back to 29.60 Ma, which was slightly earlier than other studies (Chassot et al., 2001; Von Hagen and Kadereit, 2002; Cao et al., 2021). Geologic evidence demonstrated that the turn of the Oligocene and Miocene was a crucial period of the tectonic evolution of the Qinghai–Tibetan Plateau(QTP), the central part of the QTP rose to a height of nearly 3,000 m in the Early Miocene, the cooling effect made by QTP uplift resulted in the transition of QTP from tropical and subtropical environment to a warm and cool environment consistent with the temperate climate, and the further development of herbaceous plants began in the Early Miocene (Deng et al., 2019). During this period, a primitive group of *Swertia* L. plants appeared, represented by subgen. *Swertia* L., which was characterized by perennial herbs, single stems, unbranched, and large but few flowers (Cao et al., 2021).

During the following 20 Ma to 10 Ma period, the QTP was further uplifted, and the Himalayan mountains and Tianshan Mountains were significantly elevated, which strongly changed the atmospheric circulation. Meanwhile, the global temperature decreased from the optimum temperature in the middle Miocene of the third century, resulting in a cool and dry climate (Miao et al., 2012). During this period, *Swertia* L. plants appeared as annual herbs with strongly branched stems and many small flowers, represented by subgen. *Ophelia*. The new taxa produced a large number of seeds during their life cycle and thus were better adapted to changing environments (Cao et al., 2021). When the climate was suitable, the new species gave rise to a large number of offspring, which has the potential for a great deal of variation.

Since 10 Ma, the QTP has been further uplifted in the late Miocene and Pliocene periods, and the Himalayas have blocked almost all the warm and wet air masses from the Indian Ocean, and the QTP has become cold and arid. Since 4 Ma, the QTP has been affected by Quaternary glaciation (Li et al., 1999; Mulch et al., 2006). The complex landform and rapidly changing climate resulted in many isolated small populations of *Swertia* L. which underwent radiation differentiation due to differentiated selection and random factors, forming new species adapted to local environment in a relatively short period of time. This process of radiation differentiation eventually led to the diversity of *Swertia* L. plants today. In this study, 13 species of the 23 *Swertia* L. species were formed at Quaternary. This group is the most richly differentiated and most widely adapted in *Swertia* L. with distribution in both plateau and plain.

## Conclusion

The chloroplast genome lengths of 23 species of *Swertia* L. were between 149,036 bp and 153,691 bp. The chloroplast genomes of *Swertia* L. contained 134 genes: eight rRNA, 38 tRNA, and 88 protein-coding genes. Introns were found in five tRNA and 11 protein-encoding genes. The chloroplast genomes of the 23 species of *Swertia* L. contained interspersed repeat sequences and tandem repeat sequences. The IR region variability was significantly inferior to that of the LSC and SSC regions. The majority of the protein-coding genes were comparatively well-conserved, expect for *rpoC1*, *ccsA*, *ndhI*, *ndhA*, and *rps15*, which had high variation and could potentially serve as DNA molecular barcodes. The highly differentiated regions were generally located in intergenic regions. *Swertia* L. was found to not be monophyletic, and the division of subgen. *Swertia* and subgen. *Ophelia* was supported by molecular data. However, the molecular data only partly supported the division of sect. *Ophelia*, sect. *Platynema*, sect. *Poephila,* sect*. Swertia*, and sect. *Macranthos.* The systematic positions of other groups and species require further investigation. The *Swertia* L. formed at 29.60 Ma. Speciation of 10 species occurred in succession after 12 Ma and 13 species occurred in succession after 2.5 Ma.

## Data Availability

The datasets presented in this study can be found in online repositories. The names of the repository/repositories and accession number(s) can be found in the article/Supplementary Material.
