# Supplementary material for: Comparative chloroplast genome analyses of 23 species in Swertia L. (Gentianaceae) with implications for its phylogeny
Source: Front Genet. 2022 Aug 31;13:895146. doi: 10.3389/fgene.2022.895146 (PMC9470856; doi:10.3389/fgene.2022.895146)
Supplement: Supplementary file 4 [file DataSheet1.docx]

Neupane, S., Sijapati, J., Bhattarai, T., Shrestha, S. (2020). Genetic diversity in Nepalese population of *Swertia chirayita* (Roxb. Ex Fleming) H. Karst based on inter-simple sequence repeats (ISSR) markers. *Planta*. *Afr. J. Biomed. Res.* 16(16), 895-907.

Chhipi Shrestha J. K., Bhattarai, T., Sijapati, J., Rana, N., Maharjan, J., Rawal, D. S., Raskoti, B. B., Shrestha, S. (2013). Assessment of Genetic Diversity in Nepalese Populations of *Swertia chirayita* (Roxb. Ex Fleming) H. Karst Using RAPD-PCR Technique. *Am. J. Bot.* 4, 1617-1628.

Beier, S., Himmelbach, A., Colmsee, C., Zhang, X. Q., Barrero, R. A., Zhang, Q., Barrero, R.A., Zhang, Q.S., Li, L., Bayer, M., Bolser, D., Taudien, S., Groth, M., Felder, M., Hastie, A., Šimková, H., Staňková, H., Vrána, J., Chan, S., Muñoz-Amatriaín, M., Ounit, R., Wanamaker, S., Schmutzer, T., Aliyeva-Schnorr, L., Grasso, S., Tanskanen, J., Sampath, D., Heavens, D., Cao, S.J., Chapman, B., Dai, F., Han, Y., Li, H., Li, X., Lin, C.Y., McCooke, J.K., Tan, C., Wang, S.B., Yin, S.Y., Zhou, G.F., Poland, J.A., Bellgard, M.I., Houben, A., Doležel, J., Ayling, S., Lonardi, S., Langridge, P., Muehlbauer, Gary.J., Kersey, P., Clark, M.D., Caccamo, M., Schulman, A.H., Platzer, M., Close, T.J., Hansson, M., Zhang, G.P., Braumann, I., Li, C.D., Waugh, R., Scholz, U., Stein, N., Mascher, M. (2017). Construction of a map-based reference genome sequence for barley, *Hordeum vulgare* L. *Sci. Data* 4, 170044.

McNeal, J.R., Leebens-Mack, J.H., Arumuganathan, K., Kuehl, J.V., Boore, J.L., DePamphilis, C.W. (2006). Using partial genomic fosmid libraries for sequencing complete organellar genomes. *BioTechniques* 41(1), 69-73.

Diekmann, H., Anichtchik, O., Fleming, A., Futter, M., Goldsmith, Paul., Roach, A., Rubinsztein, D.C. (2009). Decreased BDNF levels are a major contributor to the embryonic phenotype of huntingtin knockdown zebrafish. *J. Neurosci*. 29(5), 1343-1349.

Casas-Vargas, A., Romero, L.M., Usaquén, W., Zea, S., Silva, M., Briceño, I., Gómez, A., Rodríguez, J.V. (2017). Mitochondrial DNA diversity in Prehispanic bone remains on the Eastern Colombian Andes. *Biomedica* 37, 548-560.
